# Heat shock protein 90α may serve as a biomarker for mild cognitive impairment in type 2 diabetes mellitus patients without diabetic nephropathy

**DOI:** 10.3389/fimmu.2025.1516975

**Published:** 2025-05-12

**Authors:** Hui Zhang, Meiyan Chi, Songtao Feng, Wenwen Zhu, Hongxiao Wang, Wan Zhou, Bing Song, Wei Wang, Haoqiang Zhang

**Affiliations:** ^1^ Luoyang Key Laboratory of Clinical Multiomics and Translational Medicine, Henan Key Laboratory of Rare Diseases, Endocrinology and Metabolism Center, The First Affiliated Hospital, and College of Clinical Medicine of Henan University of Science and Technology, Luoyang, China; ^2^ Department of Endocrinology, Anhui Provincial Hospital Affiliated to Anhui Medical University, Hefei, China; ^3^ Department of Endocrinology, Centre for Leading Medicine and Advanced Technologies of IHM, The First Affiliated Hospital of USTC, Division of Life Sciences and Medicine, University of Science and Technology of China, Hefei, China; ^4^ Department of Nephrology, Jiangsu University Affiliated People’s Hospital, Zhenjiang, China; ^5^ Department of Endocrinology, Huzhou Central Hospital, Affiliated Central Hospital of Huzhou University, Fifth School of Clinical Medicine of Zhejiang Chinese Medical University, Huzhou, China; ^6^ Department of Endocrinology, The First Affiliated Hospital of Jinzhou Medical University, Jinzhou, China

**Keywords:** heat shock protein 90α, mild cognitive impairment, diabetic nephropathy, type 2 diabetes mellitus, oxidative stress, inflammation

## Abstract

**Aim:**

Chronic inflammation associated oxidative stress is a key factor in complications of type 2 diabetes mellitus (T2DM), including mild cognitive impairment (MCI), partly associated with cerebrovascular lesions including both macrovascular and microvascular changes, and diabetic nephropathy (DN), a kind of diabetic microvascular complication. Heat shock protein 90α (Hsp90α) is known to play a significant role in inflammation associated oxidative stress and DN. This study aims to explore the role of Hsp90α in MCI and its potential as a diagnostic marker for MCI in T2DM patients.

**Methods:**

We included 119 T2DM patients and analyzed their clinical data, Hsp90α levels, and cognitive scores. The relationships among Hsp90α, cognitive function, and urinary albumin-to-creatinine ratio (UACR) were also examined. Binary logistic regression was used to identify MCI risk factors, and ROC curves assessed Hsp90α’s diagnostic value for MCI in patients, with or without DN.

**Results:**

Patients with MCI exhibit worse cognitive function, higher UACR, and elevated Hsp90α levels compared to those without MCI. Increased Hsp90α was linked to lower cognitive scores and was identified as a risk factor for MCI. Patients with DN had a higher rate of MCI and cognitive decline than those without DN, and Hsp90α levels correlated with UACR, a DN marker. In patients without DN, higher Hsp90α was a risk factor for MCI; however, this was not observed in those with DN. An Hsp90α cut-off point of 69.105 ng/mL had a sensitivity of 60.0% and specificity of 91.4% for predicting MCI in patients without DN.

**Conclusions:**

Elevated Hsp90α level is a risk factor for cognitive impairment and may serve as a biomarker for MCI in T2DM patients without DN.

## 1 Introduction

The mechanisms underlying cognitive dysfunction in diabetes are associated not only with neurodegenerative changes related to insulin resistance (1) and hyperglycemia (2, 3) but also with cerebrovascular events related to hypoglycemia (4, 5), other cerebrovascular lesions including both macrovascular (6) and microvascular (7) changes), neuroinflammation, and oxidative stress. chronic inflammation associated oxidative stress is one of the key mechanisms underlying the onset and progression of diabetes and is widely present in both high-risk populations and individuals with diabetes (8, 9). It also plays a crucial role in the development of diabetic complications, such as diabetic nephropathy (DN), one of the microvascular complications of diabetes (10), and diabetic cognitive dysfunction, particularly in patients with type 2 diabetes mellitus (T2DM), where it accelerates the occurrence and progression of these complications. The body maintains a relatively balanced state of oxidative stress and inflammation due to the coexistence of oxidative and antioxidative mechanisms (11, 12). However, when this balance is disrupted, metabolic disorders gradually develop, affecting not only diabetes (13) itself but also its complications, including DN (14) and cognitive dysfunction (15–17).

For diabetes, oxidative stress and inflammation not only leads to the development of diabetes and its complications, but the metabolic disorder state following the onset of diabetes and its complications is also an important factor that exacerbates oxidative stress and inflammation (18–20). Taking DN as an example, oxidative stress and inflammation play roles in its occurrence and progression. As DN progresses, the increase in reactive oxygen species (ROS) and inflammation factors in kidney tissue not only causes damage to the kidneys themselves but may also lead to damage to other organs (21, 22). However, due to the body’s antioxidant mechanisms, such as heat shock proteins (HSPs), these mechanisms are activated to maintain a relative balance in inflammation associated oxidative stress as ROS and inflammation factors levels increase (23, 24). This is crucial for limiting the damage oxidative stress and inflammation can cause to the body. Indeed, in previous studies, heat shock protein 90α (Hsp90α), the most extensively studied, has been shown to play a significant role in DN (25, 26) and other diabetic complications (27).

Although compensatory increases in HSPs play a crucial role in protecting the body against oxidative stress and inflammation, excessive elevation of these proteins may cause damage to certain tissues. In kidneys, for instance, while an increase in Hsp90α can protect against inflammation associated oxidative stress, it may also contribute to kidney damage by promoting fibrosis (28–30), leading to further deterioration of renal function. However, in organs where fibrosis is not a primary mechanism underlying diabetic complications, elevated Hsp90α levels may predominantly exert protective effects. For the central nervous system, increased Hsp90α might primarily help safeguard against inflammation associated oxidative stress, thereby reducing the risk of mild cognitive impairment (MCI) in patients with T2DM.

Some studies suggest that, independent of diabetes, changes in serum Hsp90 expression were previously identified in MCI patients compared to controls (31). Hsp90 is known to be associated with neurodegenerative diseases. It has been suggested that the expression of H Hsp90 may contribute to the cognitive decline observed in patients with Alzheimer’s disease and Parkinson’s disease (32). However, the role of Hsp90 involved in cognitive impairment in patients with T2DM is still unclear. Based on the above assumptions, we believe that inflammation associated oxidative stress may lead to cognitive impairment in diabetic patients, and an adaptive increase in Hsp90α could be detected in peripheral blood. It may serve as a potential peripheral biomarker for MCI in diabetes. On the other hand, in the context of DN, the ROS and inflammation factors produced by inflammation associated oxidative stress in the kidneys could further contribute to cognitive impairment. Considering the crucial role of renal fibrosis in the development and progression of DN, as well as the influence of Hsp90 on fibrosis-related signaling pathways, we hypothesize that the accuracy of Hsp90 as a peripheral blood marker for MCI in diabetes may vary between individuals with and without DN. Therefore, in this study, we aim to verify the potential impact of DN on cognitive function in patients with type 2 diabetes; to explore the relationship between Hsp90α and MCI in individuals without DN; and to assess the value of Hsp90 as a biomarker for MCI in patients with T2DM.

## 2 Methods

### 2.1 Study design and ethical approval

A total of 119 patients from the Department of Endocrinology, The First Affiliated Hospital of USTC, all diagnosed with T2DM, were enrolled in the study. Among them, 48 patients were diagnosed with MCI and assigned to the MCI group, while the remaining 71 patients with normal cognitive function were placed in the control group. Before joining the study, all participants were given detailed information about the study’s objectives and procedures, and they provided informed consent by signing a consent form. This study received ethical approval from the Ethics Committee for Medical Research at The First Affiliated Hospital of USTC (Approval No.: 2023-RE-292).

### 2.2 Inclusion and exclusion criteria

All participants in the study were diagnosed with diabetes based on the World Health Organization’s 1999 criteria (33), with each having lived with the condition for more than three years. Out of the individuals enrolled, 48 were identified as having MCI following the guidelines set by the MCI Working Group of the European Consortium on Alzheimer’s Disease (34). The remaining 71 participants did not meet the MCI criteria but were classified in the Non-MCI group as they met the requirements for T2DM. Additionally, the diagnosis of diabetic nephropathy was made by clinicians in the medical history system. The exclusion criteria mirrored those from a previous study (35). Due to the significant variability in blood test results among patients with severe renal impairment, individuals with T2DM and severe renal impairment were excluded from the study.

### 2.3 Clinical data

The study collected clinical data from patients, including age and gender as well as diabetes and hypertension duration. Upon admission, anthropometric measurements such as weight and height were recorded, and body mass index (BMI) was calculated using the formula: weight (kg)/height (m)^2^. On the second day of hospitalization, blood samples were taken to measure levels of glycated hemoglobin (HbA1c), serum uric acid (SUA), creatinine (Cr), and blood urea nitrogen (BUN) at the Central Laboratory of the first affiliated hospital of USTC. Morning urine samples were also collected from patients to detect microalbuminuria and urinary creatinine, which were then used to calculate the Urine albumin-to-creatinine ratio (UACR). All data were obtained from the patients’ medical records. Details on the method used to determine Hsp90α levels are provided below.

### 2.4 Neurophysiological tests

All neuropsychological tests were described as our previous study (36–38). This study assessed overall cognitive function using the MoCA scores. For participants with less than 12 years of education, an extra point was added to the MoCA score in line with established guidelines. The processing speed of information was measured using the Trail Making Test A (TMTA), as previously described. Executive function was evaluated with the Digit Span Test (DST), Verbal Fluency Test (VFT), and Trail Making Test B (TMTB), following methods used in earlier studies. Auditory verbal learning test-immediate recall (AVLT-IR) and auditory verbal learning test-delayed recall (AVLT-DR) were conducted to exam the instantaneous memory function and delayed memory function, respectively. Additionally, scene memory was evaluated through the Logical Memory Test (LMT).

### 2.5 Measurement of Hsp90α

Apart from the previously mentioned measurements, we also collected and processed blood samples to obtain plasma. The blood samples were centrifuged at 4°C for 30 minutes at a relative centrifugal force of 1000 g. The plasma obtained was then analyzed for Hsp90α concentration using a Quantitative Detection Kit specifically designed for Heat Shock Protein 90α, following the protocol provided by the manufacturer (Yantai Protgen Biotechnology Development Co. Ltd, Yantai, China).

### 2.6 Statistical methods

Taking into account the prevalence of MCI in population, a Phi coefficient of 0.2 for the correlation between risk factors, and setting α at 0.05 and β at 0.10, we used to estimate the required sample size for both the case and control groups by PASS 11 software. Data analysis was conducted using SPSS version 22.0 (IBM, USA). For normally distributed variables, such as LDL-C and BUN, the differences between the two groups were presented as means with standard deviations and assessed using Student’s t-tests. For variables that were not normally distributed, including age, DM duration, BMI, HbA1c, TG, TC, HDL-C, SUA, Cr, BUN, eGFR, UACR, Hsp90α, MoCA, DST, VFT, TMTA, TMTB, AVLT-IR, AVLT-DR, and LMT, the differences were expressed as medians with interquartile ranges and analyzed using the nonparametric Mann–Whitney U test. Gender was treated as a categorical variable, described using frequency and percentage, and its differences between groups were evaluated by the chi-squared test. Pearson and partial correlation analyses were conducted with and without adjustments for factors, respectively. Furthermore, binary logistic regression analysis was employed to determine risk factors for MCI. Finally, receiver operating characteristic (ROC) curves were used to determine the optimal cut-off points and diagnostic values for all patients (both with and without DN).

## 3 Results

### 3.1 Comparison of clinical characteristics, Hsp90α levels and cognitive preference in T2DM patients with and without MCI

In this study, we first compared the clinical characteristics of T2DM patients with MCI and those without MCI. Although this was a cross-sectional study and the two groups were not strictly matched, our data showed no significant differences in age, sex, or duration of diabetes between the 2 groups. Moreover, several relevant factors, including BMI, and levels of HbA1c, FPG, TG, TC, HDL-C, LDL-C, SUA, Cr, BUN, and eGFR, were similarly distributed across both groups (all P > 0.05). This clinical section of the study aimed to explore the potential involvement of Hsp90α in T2DM patients with MCI. To this end, we conducted a comparative analysis of plasma Hsp90α levels and cognitive performance scores (assessed using the MoCA, DST, VFT, TMTA, TMTB, AVLT-IR, AVLT-DR, and LMT) between T2DM patients with and without MCI. Our findings revealed significantly higher levels of Hsp90α in patients with cognitive impairment compared to those with normal cognition (P = 0.033). Additionally, patients with MCI had lower scores on the MoCA, DST, VFT, AVLT-IR, AVLT-DR, and LMT, and higher scores on the TMTA and TMTB, compared to those without MCI (all P < 0.05) (see **Table 1**).

**Table 1:** Comparation of clinical parameters and neurophysiological test results between Non-MCI group and MCI group.

	Non-MCI (n=71)	MCI (n=48)	P
Age (year)	65 (56, 70)	65 (59, 74.50)	0.787^a^
Female (n, %)	33, 46.48	24, 50.0	0.706^c^
Diabetes duration (year)	10.00 (6.00, 19.00)	10.00 (8.50, 20.00)	0.780 ^a^
Hypertension duration (year)	0.00 (0.00, 10.00)	4.50 (0.00, 10.00)	0.046 ^a*^
HbA1c (%)	7.50 (6.90, 8.70)	8.00 (7.15, 9.65)	0.167 ^a^
BMI (kg/m^2^)	23.67 (21.94, 26.48)	24.10 (21.45, 26.18)	0.793^a^
SUA (umol/l)	305.40 (256.80, 366.60)	317.00 (267.50, 369.60)	0.525 ^a^
Cr (umol/l)	62.00 (53.00, 74.00)	66.00 (51.00, 82.75)	0.313 ^a^
BUN (mmol/l)	6.23 ± 1.66	6.69 ± 2.19	0.063 ^b^
Cr/BUN	25.20 (20.44, 27.60)	22.63 (16.86, 31.63)	0.390 ^a^
eGFR (ml/min/1.73 m^2^)	95.66 (86.87, 101.68)	90.55 (71.11, 101.20)	0.108 ^a^
UACR (mg/g)	10.49 (6.32, 22.04)	74.72 (32.09, 235.58)	<0.001 ^a*^
Hsp90α (ng/ml)	41.28 (32.63, 51.52)	50.73 (39.07, 75.20)	0.033 ^a*^
MoCA	27.00 (26.00, 28.00)	24.00 (23.00, 25.00)	<0.001 ^a*^
DST	13.00 (11.00, 17.00)	10.50 (7.00, 13.00)	0.003 ^a*^
VFT	16.00 (14.00, 19.00)	12.00 (9.25, 14.00)	<0.001 ^a*^
CDT	3.00 (2.00, 4.00)	2.00 (2.00, 3.00)	0.003 ^a*^
TMTA	59.00 (47.00, 76.00)	79.00 (62.00, 89.75)	0.012 ^a*^
TMTB	158.00 (139.00, 193.00)	192.00 (153.00, 213.50)	0.012 ^a*^
AVLT-IR	16.00 (13.00, 19.00)	13.00 (11.00, 17.00)	0.033 ^a*^
AVLT-DR	9.00 (8.00, 12.00)	8.00 (6.00, 10.00)	0.026 ^a*^
LMT	7.00 (5.00, 10.00)	5.00 (4.00, 7.75)	0.040 ^a*^

a The Mann-Whitney U test was employed for asymmetrically distributed variables.

b Student’s t test was employed for normally distributed variables.

c The Chi-square test was employed for categorical variables. ^*^P<0.05. MCI, mild cognitive impairment; BMI, body mass index; HbA1c, glycosylated hemoglobin; SUA, serum uric acid; Cr, creatinine; BUN, blood urea nitrogen; Cr/BUN, creatinine-to-blood urea nitrogen ratio; eGFR, Estimated glomerular filtration rate; UACR, Urine albumin-to-creatinine ratio, Hsp90α, Heat shock protein 90α; MoCA, Montreal cognitive assessment; DST, digit span test; VFT, verbal fluency test; CDT, clock drawing test; TMTA, trail making test-A; TMTB, trail making test-B; AVLT-IR, auditory verbal learning test-immediate recall; AVLT-DR, auditory verbal learning test-delayed recall; LMT, logical memory test.

### 3.2 Association between Hsp90α levels and cognitive preference in patients with T2DM

To explore the possible link between Hsp90α and cognitive performance in individuals with T2DM, Pearson correlation analyses were conducted. The results revealed significant negative correlations between Hsp90α levels and MoCA scores (R = -0.398, P < 0.001), and VFT (R = -0.270, P = 0.003) in T2DM patients. Moreover, partial correlation analyses were further carried out, adjusting for age, gender, diabetes duration, hypertension duration and HbA1c. Interestingly, these analyses showed negative correlations between Hsp90α levels and MoCA (R = -0.355, P < 0.001), and VFT (R = -0.252, P = 0.007) scores, after adjusting for age, gender, diabetes duration, hypertension duration and HbA1c (see **Table 2**).

**Table 2:** Partial correlation between Hsp90α and cognitive functions in patients with T2DM adjusting for age and gender as well as diabetes, hypertension duration and HbA1c.

	Model 1		Model 2	
	R	P	R	P
MoCA	-0.398	<0.001^*^	-0.355	<0.001^*^
DST	-0.172	0.062	-0.179	0.057
VFT	-0.270	0.003^*^	-0.252	0.007^*^
CDT	-0.107	0.248	-0.112	0.237
TMTA	0.157	0.127	0.149	0.113
TMTB	0.133	0.148	0.129	0.170
AVLT-IR	-0.108	0.243	-0.087	0.358
AVLT-DR	-0.081	0.384	-0.057	0.546
LMT	-0.092	0.318	-0.051	0.586

Model 1 showed the Pearson association between Hsp90α and Kidney function in patients with T2DM; Model 2 showed the partial correlation between Hsp90α and Kidney function adjusting for age, gender, diabetes duration, hypertension duration and HbA1c.

^*^P<0.05. Hsp90α, Heat shock protein 90α; T2DM, type 2 diabetes mellitus; MoCA, Montreal cognitive assessment; DST, digit span test; VFT, verbal fluency test; CDT, clock drawing test; TMTA, trail making test-A; TMTB, trail making test-B; AVLT-IR, auditory verbal learning test-immediate recall; AVLT-DR, auditory verbal learning test-delayed recall; LMT, logical memory test.

### 3.3 Analysis for the risk factor for MCI in patients with T2DM

Since Hsp90α levels are linked to MoCA scores, which reflect overall cognitive function, we hypothesized that Hsp90α might be a potential risk factor for MCI in individuals with T2DM. To investigate whether elevated Hsp90α levels could serve as a risk factor for MCI among T2DM patients, we conducted a binary logistic regression analysis. The findings revealed that higher plasma levels of Hsp90α are indeed associated with an increased risk of MCI in T2DM patients (OR = 1.032, P = 0.002). Furthermore, even after adjusting for age, gender, diabetes duration, hypertension duration and HbA1c, increased Hsp90α levels continued to be identified as a risk factor for MCI in these patients (OR = 1.037, P = 0.001) (**Supplementary Table 1**).

### 3.4 Difference of the relationship between Hsp90α and MCI in T2DM patients with and without DN

Based on previous basic research, it was found that Hsp90α might be closely associated with DN. In this study, we found that the UACR levels in patients with MCI were significantly higher than those in patients without MCI (p < 0.001) (**Table 1**). Since UACR is an important marker of DN, we subsequently analyzed the correlation between renal function and Hsp90α. Indeed, a correlation was found only between Hsp90α and UACR with and without adjusting for age, gender, diabetes duration, and hypertension duration (**Table 3**). This suggests a close and complex relationship between Hsp90α, DN, and MCI. Further analysis revealed that, compared to 68 T2DM patients without DN, 58 patients with T2DM and DN exhibited higher incidence of MCI (p<0.001), as well as lower MoCA scores (p<0.001) (reflecting overall cognitive function) and VFT scores (p<0.001), (reflecting executive function) (**Table 4**). Due to the complex relationship among these three factors, we conducted further subgroup analyses to explore the relationship between Hsp90α and MCI. The subgroup analysis showed that in T2DM patients without DN, elevated Hsp90α was an independent risk factor for MCI in T2DM patients, regardless of whether age, gender, diabetes duration, and hypertension duration were adjusted (OR=1.065, P=0.001; OR=1.067, P=0.003). However, in diabetic patients with DN, the result was negative (OR=1.024, P=0.073; OR=1.028, P=0.135) (**Supplementary Table 2**).

**Table 3:** Association between Hsp90α and kidney functions in patients with T2DM.

	Model 1		Model 2	
	R	P	R	P
SUA	0.101	0.272	0.083	0.378
Cr	0.077	0.405	0.022	0.813
BUN	0.026	0.783	-0.021	0.820
Cr/BUN	-0.085	0.355	-0.079	0.406
eGFR	-0.058	0.531	-0.028	0.771
UACR	0.372	<0.001^*^	0.362	<0.001^*^

Model 1 showed the Pearson association between Hsp90α and Kidney function in patients with T2DM; Model 2 showed the partial correlation between Hsp90α and Kidney function adjusting for age, gender, diabetes duration, hypertension duration and HbA1c. *P<0.05. Hsp90α, Heat shock protein 90α; SUA, serum uric acid; Cr, creatinine; BUN, blood urea nitrogen; Cr/BUN, creatinine-to-blood urea nitrogen ratio; eGFR, Estimated glomerular filtration rate; UACR, Urine albumin-to-creatinine ratio.

**Table 4:** Comparation of neurophysiological test results between Non-DN group and DN group.

	Non-DN (n=68)	DN (n=51)	P
MCI (n, %)	10, 14.71%	38, 74.51%	<0.001^*^
MoCA	27.00 (26.00, 28.00)	25.00 (24.0, 26.00)	<0.001^*^
DST	13.00 (11.00, 17.00)	11.00 (8.00, 14.00)	0.116
VFT	16.00 (13.00, 19.00)	12.00 (11.00, 15.00)	<0.001^*^
CDT	3.00 (2.00, 3.00)	2.00 (2.00, 3.00)	0.106
TMTA	62.50 (49.00, 78.25)	73.00 (56.00, 85.00)	0.412
TMTB	166.00 (138.25, 203.75)	186.00 (152.00, 210.00)	0.118
AVLT-IR	16.00 (13.00, 19.00)	14.00 (11.00, 17.00)	0.254
AVLT-DR	9.00 (7.00, 12.00)	8.00 (6.00, 11.00)	0.246
LMT	7.00 (4.25, 10.00)	6.00 (4.00, 8.00)	0.068

^*^P<0.05; DN, diabetic nephropathy; MCI, mild cognitive impairment; MoCA, Montreal cognitive assessment; DST, digit span test; VFT, verbal fluency test; CDT, clock drawing test; TMTA, trail making test-A; TMTB, trail making test-B; AVLT-IR, auditory verbal learning test-immediate recall; AVLT-DR, auditory verbal learning test-delayed recall; LMT, logical memory test.

### 3.5 Evaluation the diagnosis values of Hsp90α for MCI in patients with T2DM

**Figure 1:**
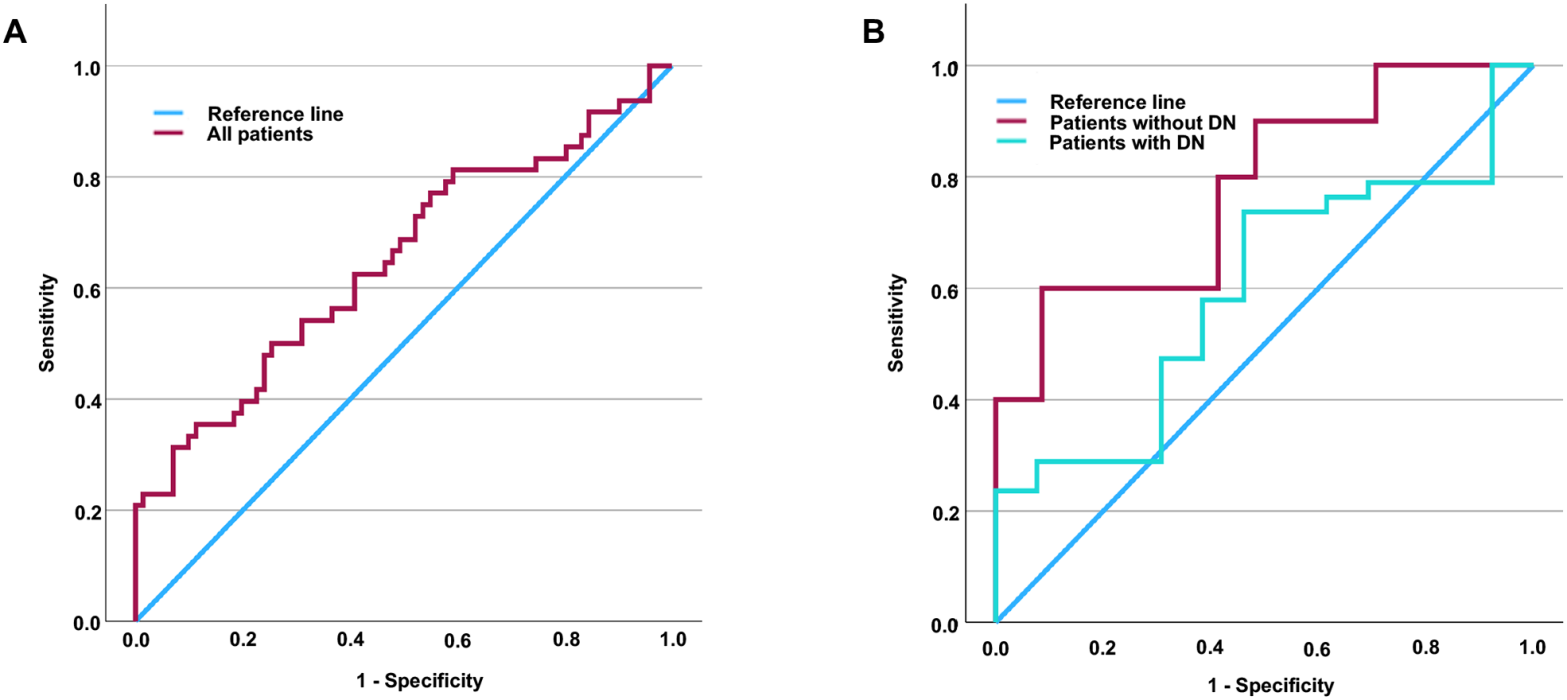
ROC curve of Hsp90α for the sensitivity and specificity of MCI in patients with T2DM. The area under the curve is 0.656 in all patients **(A)**. Additionally, the area under the curve is 0.597 and 0.781 in patients with and without DN, respectively **(B)**. It is determined that the diagnostic cut-off value for Hsp90α to be 69.105 pg/ml, revealed a sensitivity of 60.0% and specificity of 91.4% in T2DM patients without DN (Youden’s index=0.514). ROC, Receiver operating characteristic; Hsp90α, Heat shock protein 90α; MCI, mild cognitive impairment; T2DM, type 2 diabetes mellitus; DN, diabetic nephropathy; Hsp90α, Heat shock protein 90α.

Given that an elevated level of Hsp90α has been recognized as a potential risk factor for MCI in patients with T2DM, we undertook a comprehensive evaluation of Hsp90α’s diagnostic value in all patients with T2DM. ROC curve analysis showed an area under the curve (AUC) of 0.656 in all T2DM patients (see **Figure 1A**). As previously noted, the relationship between Hsp90α and MCI differs significantly between patients with and without DN. Therefore, we further assessed the diagnostic value of Hsp90α for MCI specifically in patients without DN. We determined that the diagnostic cut-off value for Hsp90α is 69.105 ng/ml. Additionally, our analysis demonstrated a sensitivity of 60.0%, a specificity of 91.4% (Youden’s index=0.514), and an AUC of 0.781 for this cut-off value (refer to **Figure 1B**).

## 4 Discussion

To our best knowledge, while many studies have previously explored the relationship between oxidative stress and cognitive function (39), some have also preliminarily investigated the potential link between HSPs, closely related to oxidative stress (40, 41), and cognitive function (42). This study is the first to examine the relationship between oxidative stress-related Hsp90α and cognitive dysfunction in individuals with T2DM. We initially compared the basic characteristics of T2DM patients with and without MCI. Although, as a cross-sectional study, we did not strictly match the two groups for age, sex, and diabetes duration, these factors did not show significant differences between them. Moreover, while plasma glucose control levels are associated with cognitive dysfunction, we found that T2DM patients with cognitive dysfunction had slightly elevated HbA1c levels reflecting plasma glucose control, although this was not statistically significant. This aligns with previous findings was found (37, 38, 43, 44), as hyperglycemia, plasma glucose fluctuations, and hypoglycemia can all contribute to cognitive dysfunction. Therefore, differences in plasma glucose control levels between the two groups may exist across different populations. Indeed, plasma glucose control is closely related to diabetes-related complications. In our previous studies, we found that, compared to the control group, patients with diabetic peripheral neuropathy had higher HbA1c levels (45–49), and similarly, patients with cognitive dysfunction also had higher HbA1c levels compared to the control group (35, 36). However, some of our studies have results similar to the current one. Although cognitive dysfunction in diabetic patients showed higher HbA1c levels compared to the control group, no statistical difference was found (37, 38, 43). As we described in the introduction of our manuscript, poor blood glucose control may contribute to the development of cognitive dysfunction, but hypoglycemic events also represent a risk factor for cognitive impairment in diabetes. Although we excluded patients with severe hypoglycemic events in our study, we cannot rule out the possibility of undetected hypoglycemia. The proportion of such patients varies across studies, which may explain the differing results in different studies. To account for the impact of plasma glucose levels, we adjusted for HbA1c when analyzing the relationship between HSP90α and cognitive function. Uric acid, a natural antioxidant, was also a focus of our study due to its relation to oxidative stress (50). We compared serum uric acid levels between the two groups and found no significant differences. Previous research suggested a U-shaped correlation between uric acid levels and cognitive function, where excessively low levels may lead to cognitive impairment, while moderate levels could provide neuroprotective effects due to their antioxidant properties. Conversely, high uric acid levels may mask its protective effects due to metabolic disturbances affecting cognition (51). Indeed, the neuroprotective role of uric acid may extend beyond the central nervous system; our recent studies found that low uric acid levels are associated with risk factors for diabetic peripheral neuropathy, while elevated levels may protect peripheral nerves, as T2DM patients with high uric acid exhibited faster nerve conduction velocities (46).

In the overall population, we found that patients with T2DM who also presented with cognitive impairment exhibited elevated levels of Hsp90α. Further Pearson correlation analysis revealed a negative correlation between Hsp90α and both the MoCA score, which reflects global cognitive function, and the VFT score, which reflects executive function. Although we identified differences in the duration of hypertension between T2DM patients with and without MCI, there were no significant differences in age, gender, duration of diabetes, and levels of HbA1c. In our further correlation analyses, we not only adjusted for the duration of hypertension but also for age, gender, duration of diabetes and levels of HbA1c to mitigate the limitations inherent in our cross-sectional study design. Interestingly, even after adjusting for these factors, partial correlation analysis still indicated a negative correlation between Hsp90α and both the MoCA and VFT scores. To further validate the relationship between Hsp90α and cognitive impairment, we conducted logistic regression analyses, which revealed that elevated Hsp90α is an independent risk factor for MCI in T2DM patients, regardless of whether we adjusted for age, gender, duration of diabetes, or duration of hypertension. There are many diagnostic methods for assessing cognitive dysfunction. Currently, diagnosing MCI involves a series of complex cognitive assessments that are both time-consuming and inherently subjective (52). While neuroimaging studies (including the use of PET-MRI to analyze central nervous system Aβ, Tau, and neuroinflammation) can reveal certain cognitive-related changes, their diagnostic criteria are difficult to quantify and can be quite costly (53, 54). Although some research has examined the link between imaging changes and cognitive impairment, much work remains to establish a definitive diagnosis of cognitive dysfunction. Certain biomarkers, such as those found in cerebrospinal fluid, may be relevant to cognition; however, their invasive nature presents significant risks to patients, limiting their broader use (55, 56). In contrast, peripheral blood biomarkers offer a promising alternative. They can be collected through routine clinical procedures with minimal trauma to patients, making them more acceptable. Compared to existing research methods, detecting Hsp90α in peripheral blood is an easier and more cost-effective approach (since imaging methods are often expensive and difficult to implement due to the lack of equipment in many hospitals), and it involves less trauma (as cerebrospinal fluid collection is not required, whereas many biomarkers necessitate cerebrospinal fluid collection). Due to the presence of the blood-brain barrier, peripheral blood biomarkers inherently face disadvantages compared to specimens from the central nervous system. However, peripheral blood biomarkers offer significant advantages in terms of accessibility. The findings of this study highlight the value of Hsp90α in blood as a marker for cognitive dysfunction. This can be attributed to three main reasons. First, peripheral blood and the central nervous system share similar environments, suggesting a potential correlation between the levels of factors in both areas. Second, in diabetic patients, the blood-brain barrier is compromised, allowing factors produced in the central nervous system to be detected in peripheral blood. Simultaneously, factors produced peripherally may influence the central nervous system through the impaired blood-brain barrier, contributing to cognitive dysfunction for oxidative stress (57–59). Third, certain structures, such as exosomes (60), can carry specific factors from the periphery into the central nervous system, impacting cognitive function. During episodes of cognitive dysfunction, factors produced by the central nervous system can also be transported by these structures across the blood-brain barrier into peripheral blood, where they can be detected.

Earlier, we noted that lower levels of Hsp90α in peripheral blood correlate with overall cognitive function in patients and are a risk factor for MCI in those with Type 2 Diabetes Mellitus (T2DM). Therefore, we hypothesize that decreased levels of Hsp90α in peripheral blood could serve as a biomarker for the early detection of cognitive impairment in T2DM patients. To assess the diagnostic value of Hsp90α for MCI in T2DM patients, we performed additional analysis using ROC curves, which showed an area under the curve of only 0.648.

In our analysis of the characteristics of T2DM patients with and without MCI, we not only found significant differences in the levels of Hsp90α, which is associated with oxidative stress, but also in UACR, an important marker for DN. Furthermore, we compared the cognitive functions of patients with and without DN and discovered that those with DN exhibited worse cognitive function. This was evidenced by a higher incidence of MCI, as well as lower MoCA scores, which reflect global cognitive function, and diminished VFT scores, which assess executive function. While compensatory increases in HSPs are essential for defending the body against oxidative stress and inflammation, excessive levels can have harmful effects on certain tissues. In the kidneys, for example, elevated Hsp90α may help counteract inflammation-related oxidative stress, but it can also promote fibrosis (activating inflammation and further lowing renal clearance), potentially accelerating renal damage and functional decline. In contrast, in organs where fibrosis is not a key factor in diabetic complications, higher levels of Hsp90α may primarily offer protective benefits. In the central nervous system, for instance, increased Hsp90α may mainly help shield against inflammation-induced oxidative stress, potentially lowering the risk of mild cognitive impairment MCI in individuals with T2DM. In other words, the role of Hsp90α is not exactly the same in the kidneys and the central nervous system. To clarify the relationship between Hsp90α and cognitive dysfunction in diabetes, we excluded DN in our subsequent analyses. Consequently, we explored the relationship between Hsp90α and MCI in T2DM patients with and without DN. Interestingly, in patients with DN, elevated Hsp90α was not a risk factor for MCI, irrespective of adjustments for age, gender, diabetes duration, and hypertension duration. In contrast, in patients without DN, elevated Hsp90α did serve as a risk factor for MCI, regardless of these adjustments. Due to the completely different relationship between Hsp90α and MCI in T2DM patients with and without DN, we conducted further evaluations of Hsp90α’s diagnostic value for MCI through subgroup analyses. Undoubtedly, in T2DM patients with DN, the area under the ROC curve for Hsp90α in diagnosing MCI was only 0.597. Interestingly, in T2DM patients without DN, the area under the curve of ROC reached as high as 0.781. An Hsp90α cut-off point of 69.105 ng/mL demonstrated a sensitivity of 60.0% and specificity of 91.4% for predicting MCI in patients without DN. Although the area under the ROC curve is 0.781, the sensitivity at this cut-off point (60.0%) indicates that Hsp90α has limited utility for the early detection of MCI. Nevertheless, this does not lessen the importance of the study’s findings. Given the significant psychological burden that patients experience due to fears of cognitive decline and dementia, it is crucial to be able to rule out the risk of cognitive impairment early on. Informing individuals classified as low-risk can help alleviate their concerns, allowing them to concentrate on managing their diabetes and its related complications. Our findings suggest that when peripheral blood levels of Hsp90α are below 69.105 pg/ml in T2DM patients without DN, the likelihood of developing MCI is very low. This means that such patients can avoid unnecessary anxiety about cognitive impairment and focus their efforts on diabetes treatment and management of complications.

To the best of our knowledge, no studies have yet explored the relationship between Hsp90α and cognitive dysfunction in individuals with and without DN separately. Our research has, for the first time, uncovered a link between peripheral blood Hsp90α levels and cognitive function in patients with T2DM, with implications for executive function and serving as an independent risk factor for MCI in all diabetic individuals and patients without DN. We have also highlighted its diagnostic potential as a biomarker for MCI in T2DM patients without DN. However, this study has limitations; being a cross-sectional analysis with a small sample size, we cannot definitively establish causation between Hsp90α levels and cognitive dysfunction. Further cohort studies are necessary. Nonetheless, with a larger sample size, a mediation analysis exploring the mediating role of DN (or UACR) in the effect of Hsp90a on MCI would yield even more fascinating insights. Of course, interaction effects could also be analyzed. However, due to the limited overall sample size, we did not conduct such an analysis in this study. We consider this a limitation of our research. Additionally, we did not strictly match the age, gender, duration of diabetes, or hypertension of the participants, although we adjusted for these factors in our analyses, which remains a limitation. Medication use, including diabetes treatments, may also impact cognitive function (61–63), but we only collected data on medications at enrollment without detailed dosage or duration records. Due to the small sample size, certain medications were only used by a handful of patients. As a result, a thorough analysis of medication usage isn’t feasible in our current study, which should be recognized as a limitation. However, in a separate study by our former colleagues, the potential effects of different medications on cognitive function were explored through network meta-analysis (64). Smoking has been shown to affect complications in diabetic patients (65), including neuropathy (66). In the design phase of our study, we accounted for the potential impact of smoking history on cognitive function by excluding smokers from enrollment. Similarly, individuals who consumed alcohol were also excluded (67). In addition to active smoking, exposure to secondhand smoke can also contribute to diabetic complications. However, during the course of the study, it was difficult to accurately assess the duration and intensity of patients’ exposure to secondhand smoke, which we recognize as a limitation of our research. Moreover, it would have been interesting to consider smoking intensity—such as never, former, or current smokers. However, since this was not the primary focus of our study, we addressed both active and secondhand smoke exposure as limitations in the discussion section of the manuscript. Although the results are interesting, there are still many limitations. If we were able to compare the diagnostic value of Hsp90a with other methods, or examine whether adding Hsp90a to a combined diagnostic approach could further increase the AUC, specificity, and sensitivity of the ROC curve, the results would be even more compelling.

## 5 Conclusion

In summary, we found that elevated levels of peripheral plasma Hsp90α are associated with cognitive dysfunction in patients with T2DM and act as an independent risk factor for the development of MCI in these patients. The relationship between Hsp90α and cognitive dysfunction varies significantly among T2DM patients with and without DN. Furthermore, in T2DM patients without DN, Hsp90α may serve as a biomarker for diagnosing MCI, although its effectiveness for early identification might be limited. However, low levels of Hsp90α could be valuable in ruling out MCI in T2DM patients. Clinically, it can be used as a tool for those concerned about cognitive dysfunction to exclude the presence of MCI.

## Data Availability

The original contributions presented in the study are included in the article/**Supplementary Material**. Further inquiries can be directed to the corresponding authors.

## Supplementary material

Supplementary Table 1 Assessment of risk factors for MCI in patients with T2DM by binary logistic analysis. Model 1 showed that Hsp90α is the risk factor for MCI in patients with T2DM; Model 2 showed that Hsp90α is the risk factor for MCI in patients with T2DM adjusting for age, gender, diabetes duration, hypertension duration and HbA1c; *P<0.05. MCI, mild cognitive impairment; T2DM, type 2 diabetes mellitus; Hsp90α, Heat shock protein 90α.

Supplementary Table 2 Assessment of risk factors for MCI in patients with T2DM with or without DN by binary logistic analysis. Model 1 showed that Hsp90α is the risk factor for MCI in all patients with T2DM; Model 2 showed that Hsp90α is the risk factor for MCI in patients with T2DM adjusting for age, gender, diabetes duration, hypertension duration and HbA1c; *P<0.05. MCI, mild cognitive impairment; T2DM, type 2 diabetes mellitus; DN, diabetic nephropathy; Hsp90α, Heat shock protein 90α.
