# Supplementary material for: Heat shock protein 90α may serve as a biomarker for mild cognitive impairment in type 2 diabetes mellitus patients without diabetic nephropathy
Source: Front Immunol. 2025 May 12;16:1516975. doi: 10.3389/fimmu.2025.1516975 (PMC12105644; doi:10.3389/fimmu.2025.1516975)
Supplement: Supplementary Table 1 — Assessment of risk factors for MCI in patients with T2DM by binary logistic analysis. Model 1 showed that Hsp90α is the risk factor for MCI in patients with T2DM; Model 2 showed that Hsp90α is the risk factor for MCI in patients with T2DM adjusting for age, gender, diabetes duration, hypertension duration and HbA1c; *P<0.05. MCI, mild cognitive impairment; T2DM, type 2 diabetes mellitus; Hsp90α, Heat shock protein 90α. [file Table1.docx]

Supplementary-Table 1: Assessment of risk factors for MCI in patients with T2DM by binary logistic analysis

|  | β | P | OR | 95% CI | |
| --- | --- | --- | --- | --- | --- |
|  |  |  |  | Lower | Upper |
| Model 1 | | | | | |
| Hsp90α | 0.032 | 0.002^*^ | 1.032 | 1.012 | 1.053 |
| Model 2 | | | | | |
| Hsp90α | 0.036 | 0.001^*^ | 1.037 | 1.014 | 1.060 |
| Age | 0.046 | 0.086 | 1.047 | 0.993 | 1.104 |
| Gender | -0.001 | 0.998 | 0.999 | 0.435 | 2.297 |
| Diabetes duration | -0.001 | 0.985 | 0.999 | 0.945 | 1.057 |
| Hypertension duration | 0.019 | 0.489 | 1.019 | 0.967 | 1.074 |
| HbA1c | 0.192 | 0.087 | 1.211 | 0.973 | 1.509 |

Notes: Model 1 showed that Hsp90α is the risk factor for MCI in patients with T2DM; Model 2 showed that Hsp90α is the risk factor for MCI in patients with T2DM adjusting for age, gender, diabetes duration, hypertension duration and HbA1c; ^*^ P<0.05. Abbreviations: MCI, mild cognitive impairment; T2DM, type 2 diabetes mellitus; Hsp90α, Heat shock protein 90α.

Supplementary -Table2: Assessment of risk factors for MCI in patients with T2DM with or without DN by binary logistic analysis

|  |  | β | P | OR | 95% CI | |
| --- | --- | --- | --- | --- | --- | --- |
|  |  |  |  |  | Lower | Upper |
| Non-DN | Model 1 | | | | | |
|  | Hsp90α | 0.063 | 0.001^*^ | 1.065 | 1.025 | 1.108 |
|  | Model 2 | | | | | |
|  | Hsp90α | 0.074 | 0.003^*^ | 1.076 | 1.026 | 1.129 |
|  | Age | 0.052 | 0.330 | 1.054 | 0.949 | 1.170 |
|  | Gender | 1.187 | 0.209 | 3.278 | 0.514 | 20.906 |
|  | Diabetes duration | -0.004 | 0.941 | 0.996 | 0.899 | 1.104 |
|  | Hypertension duration | 0.051 | 0.357 | 1.052 | 0.944 | 1.172 |
|  | HbA1c | 0.255 | 0.260 | 1.291 | 0.828 | 2.013 |
| DN | Model 1 | | | | | |
|  | Hsp90α | 0.023 | 0.173 | 1.024 | 0.990 | 1.059 |
|  | Model 2 | | | | | |
|  | Hsp90α | 0.028 | 0.135 | 1.028 | 0.991 | 1.066 |
|  | Age | 0.054 | 0.244 | 1.055 | 0.964 | 1.155 |
|  | Gender | -0.221 | 0.764 | 0.802 | 0.189 | 3.400 |
|  | Diabetes duration | -0.003 | 0.957 | 0.997 | 0.902 | 1.103 |
|  | Hypertension duration | -0.024 | 0.585 | 0.976 | 0.896 | 1.064 |
|  | HbA1c | 0.052 | 0.799 | 1.053 | 0.707 | 1.570 |

Notes: Model 1 showed that Hsp90α is the risk factor for MCI in all patients with T2DM; Model 2 showed that Hsp90α is the risk factor for MCI in patients with T2DM adjusting for age, gender, diabetes duration, hypertension duration and HbA1c; ^*^ P<0.05. Abbreviations: MCI, mild cognitive impairment; T2DM, type 2 diabetes mellitus; DN, diabetic nephropathy; Hsp90α, Heat shock protein 90α.
